# The Combinational Effect of Enhanced Infection Control Measures and Targeted Clinical Metagenomics Surveillance on the Burden of Endemic Carbapenem and Other β-Lactam Resistance Among Severely Ill Pediatric Patients

**DOI:** 10.3390/biomedicines13010031

**Published:** 2024-12-26

**Authors:** Athina Giampani, Maria Simitsopoulou, Maria Sdougka, Christos Paschaloudis, Emmanuel Roilides, Elias Iosifidis

**Affiliations:** 1 Infectious Disease Unit, 3rd Department of Pediatrics, School of Medicine, Faculty of Health Sciences, Hippokration General Hospital, 54642 Thessaloniki, Greece; giampani.athina1@gmail.com (A.G.); simitsop@auth.gr (M.S.); iosifidish@auth.gr (E.I.); 2 Basic and Translational Research Unit, Special Unit for Biomedical Research and Education, School of Medicine, Faculty of Health Sciences, Aristotle University of Thessaloniki, 54124 Thessaloniki, Greece; 3 Pediatric Intensive Care Unit, Hippokration General Hospital, 54942 Thessaloniki, Greece; 4 Independent Researcher, 54642 Thessaloniki, Greece; chrispashaloudis@hotmail.com

**Keywords:** targeted resistome, pediatric intensive care unit, antimicrobial resistance, enhanced infection control measures, active surveillance program, gene amplification

## Abstract

**Background:** Antimicrobial resistance (AMR) is recognized as one of the most important global public health threats. There is an urgent need to reduce the spread of these multidrug-resistant bacteria (MDR-B), particularly in extremely vulnerable patients. The aim of this study was to investigate whether targeted gene amplification performed directly on clinical samples can be used simultaneously with a bundle of enhanced infection control measures in a Pediatric Intensive Care Unit (PICU) endemic to MDR-B. **Methods**: This study had three phases: (1) the baseline phase was performed prior to intervention when first screening and sample collection were performed; (2) the intervention phase was performed when various enhanced infection control measures (EICM) were applied; and (3) the maintenance phase occurred when EICMs were combined with the implementation of targeted molecular surveillance. The presence of four carbapenemase genes, *bla*_KPC_, *bla*_OXA-48-like_, *bla*_VIM_, and *bla*_NDM_, as well as the β-lactamase genes *bla*_TEM_ and *bla*_SHV_, was evaluated by PCR after DNA isolation directly from stool samples. The results were compared to culture-based phenotypic analysis. **Results and Conclusions**: The implementation of EICM appeared to reduce the resistance burden in this sample endemic to an MDR-B clinical setting. The direct implementation of a targeted and customized rapid molecular detection assay to clinical samples seems to be an effective clinical tool for the evaluation of EICM measures.

## 1. Introduction

Antimicrobial drug resistance (AMR) is recognized as one of the most important global public health concerns [1]. It could lead to the loss of 10 million lives annually by 2050. This is on top of significant clinical and economic consequences and the quality-adjusted life years (QALYs) lost [2]. This makes AMR one of the largest challenges to global health and to healthcare systems around the world, demanding global, regional, country, and even hospital-specific collaborative solutions based on epidemiological and microbiological data. Infections caused by antimicrobial multidrug-resistant bacteria, mainly Gram-negative bacteria, in pediatric patients significantly increase morbidity, mortality, hospitalization, and costs allocated to healthcare systems worldwide [3]. The QALYs lost when infections by AMR bacteria occur in infants, and children are tremendously more than in adults. This is even more true in environments like Pediatric Intensive Care Units (PICUs) with critically ill and more or less immunocompromised patients.

Bacteria can acquire and transmit resistance through various mechanisms, among which extended spectrum β-lactamases (ESBLs) and carbapenemases are increasingly important nowadays [4]. There is an urgent need to reduce the spread of these resistant bacteria, particularly among highly susceptible patients such as critically ill infants and children, in order to save human lives. For this purpose, surveying a set of specific resistance genes in a high-risk endemic healthcare setting, in other words, searching for a targeted resistome in the hospital unit, is extremely important for understanding the resistance problem that exists in a particular hospital unit and confronting it with enhanced infection control measures and with antimicrobial stewardship programs [5]. These measures have been developed lately and can be used in individual hospitals and health regions or smaller hospitals.

We aimed to investigate the impact of various enhanced and tailored infection control measures (EICMs), including an active surveillance program with targeted gene amplification directly in clinical samples on the AMR burden in an endemic Pediatric Intensive Care Unit (PICU). Secondary objectives were a) the comparison between the direct detection of targeted AMR genes in stools and culture-based phenotypic analysis and b) the impact of enhanced and tailored infection control measures (EICMs) on healthcare workers’ compliance to hand hygiene and sampling for active surveillance, central line-associated bloodstream infection rates and antimicrobial as well as carbapenem consumption.

## 2. Materials and Methods

### 2.1. Study Design, Patient Population, and Clinical Protocol

The study was conducted in an eight-bed PICU over 18 months as part of an interventional initiative of the Infection Control Committee of Hippokration General Hospital, targeting the controlled spread of AMR genes among critically ill pediatric patients in the PICU. The study protocol was approved by the Clinical Studies Committee of the Scientific Advisory Board of Hippokration General Hospital. As the stool samples were taken routinely according to the hospital Infection Control Committee policy in the PICU, signing informed consent by the parents of the patients was deemed unnecessary.

The clinical protocol consisted of the following three phases:

(1) The baseline phase, which lasted five months, determined the existing AMR burden in the PICU. All patients hospitalized for at least 5 days in the PICU were screened for the presence of the AMR genes using PCR directly applied to stool samples. The AMR genes tested were selected based on local (hospital) and national AMR epidemiology. The presence of four carbapenemase genes, namely *bla*_KPC_, *bla*_OXA-48-like_, *bla*_VIM_, and *bla*_NDM_, as well as two other β-lactamase genes, namely *bla*_TEM_ and *bla*_SHV_, were evaluated by endpoint PCR. Patients not bearing the resistance genes under study were re-evaluated after ≥1 month of still being in the PICU for probable new colonization.

(2) The intervention phase was run for 8 months. During this time period, the EICMs were intensified through the implementation of three infection control and prevention components: (a) Enhanced compliance to routine active surveillance cultures was assessed. Weekly reminders for rectal swab collection were planned to be sent to the hospital’s microbiology department for routine surveillance cultures for AR detection, isolation, and the identification of pathogens and susceptibility testing with phenotypic assays. Sampling frequency evaluations before and after the intervention phase reflected the level of compliance with routine surveillance procedures. They were used as a measure of the process and outcome goals of the actual sampling frequency compared to the recommended sampling frequency by the hospital Infection Control and Prevention Committee. (b) Monthly educational and training meetings of the infection control and prevention team with PICU staff also took place (physicians, nurses, and rest of staff). The distribution of poster handouts and floor plans of colonized patients was also performed. (c) Audits with feedback on antimicrobial consumption and infection control were performed. The consumption of all antibiotics and carbapenems per month was expressed as the number of defined daily doses (DDDs) per 100 bed-days. Compliance with hand hygiene (direct observation, percentage of hand hygiene performances to the total number of opportunities), the consumption of antiseptic solution (liters per 100 bed-days), and CLABSI rate (number of CLABSIs per 1000 central-line days) were used to assess the implementation of these measurements.

(3) The maintenance phase of the AMR surveillance protocol lasted for another 5 months and assessed the combinational effect of two surveillance factors: the enhanced infection control measures mentioned above and the implementation of the targeted molecular panel of AMR genes customized to represent the most frequently met AMR gene burden in this specific PICU.

### 2.2. Targeted Molecular Analysis

Stool samples were collected and divided into six 0.25 g portions and stored at −80 °C until processed. DNA extraction directly from the stool samples was performed using the QIAamp^®^ PowerFecal^®^ DNA Kit (QIAGEN, Hilden, Germany) according to the manufacturer’s specifications. The AMR genes studied were the following: *bla*_KPC_, *bla*_VIM_, *bla*_NDM_, *bla*_OXA-48-like_, *bla*_TEM_, and *bla*_SHV_. AMR was assessed by endpoint PCR using the KAPA HiFi HotStart PCR Kit (Kapa Biosystems Inc, Wilmington, MA, USA), and specific primers for each targeted gene were designed with Oligo 7.0 online software (Table 1).

The thermal protocol used was the following: initial denaturation at 95 °C for 3 min, 35 cycles of 20 s denaturation at 98 °C, 15 s annealing at 55.5–61.6 °C, and 60 s of extension at 72 °C, and one cycle of 1 min final extension at 72 °C. The amplified products, with a size between 217 and 785 bp in length, were visualized on 1.5% agarose gel after adding ethidium bromide to a final concentration of 0.5 μg/mL (ApliChem GmbH, Darmstadt, Germany). Patients were found to be negative for the resistance genes under study and were re-evaluated after at least 3 weeks for probable colonization.

### 2.3. Statistical Analysis

The methodology of an interrupted time series was used to assess the impact of EICM on hand hygiene, the CLABSI ratio, and antimicrobial as well as carbapenem consumption. There were 5, 8, and 5 time points for each outcome (except for hand hygiene compliance) during the three study phases (baseline, intervention, and maintenance). Actual data and trend lines were used for the visualization of differences. The linear regression model (LRM) was used for the comparison of different phases (for each LRM, we used one predictor for the time and two predictors per intervention). Fisher’s exact test was used to compare AMR gene detection using a targeted endpoint PCR approach vs. a culture-based technique.

## 3. Results

### 3.1. Baseline, Intervention, and Maintenance Phase

**Figure 1:**
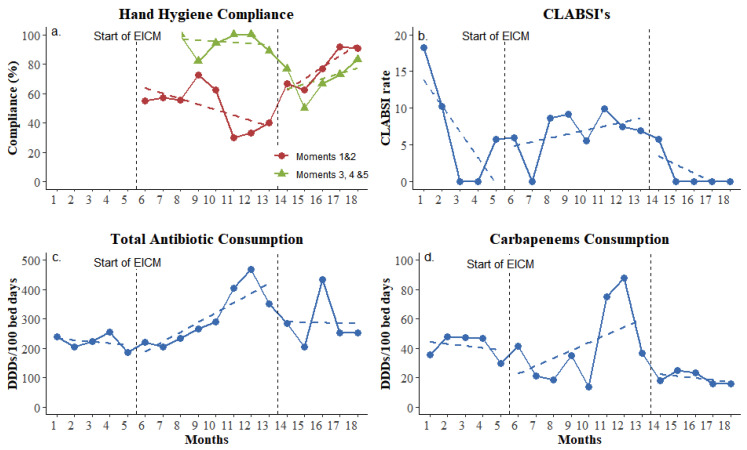
Compliance of applied enhanced infection control measurements. (**a**) Percentage of hand hygiene compliance; (**b**) CLABSI rate per 1000 central line days; (**c**) total antibiotic consumption by daily defined doses per 100 bed-days; (**d**) Carbapenem consumption by daily defined doses per 100 bed-days. EICM: enhanced infection control measures. Baseline phase: 1–5 months; intervention phase: 6–13 months; and maintenance phase: 14–18 months.

During the study period, the implementation of standard and enhanced infection control measures resulted in important changes in process goals (colonization sampling compliance) and outcome indicators (hand hygiene compliance, CLABSI ratio, and carbapenem consumption, as shown in Figure 1). Compliance with hand hygiene was at a moderate level throughout the whole study and fluctuated between 54.5% and 86.2% (Figure 1a). However, interrupted time series analysis revealed that hand hygiene before contact with the patient and before aseptic procedures (the WHO’s moments one and two) improved significantly during the maintenance phase (*p* = 0.016, change in trend according to LRM). Antiseptic solution consumption showed no significant change, but the CLABSI rate was decreased and nullified for the last four consecutive months of the study period (maintenance phase, Figure 1b). Rectal swabs taken for active surveillance cultures increased from an average of 1.45 to 6.5 per patient, showing a more than four-fold improvement.

During the intervention phase (8 months, 1469 bed-days), carbapenem’s monthly consumption decreased despite the fact that total monthly antibiotic consumption showed a small overall increase that ranged between 13.9 and 87.6 DDDs per 100 bed-days during the study period (Figure 1c,d).

### 3.2. AMR Gene Detection

**Figure 2:**
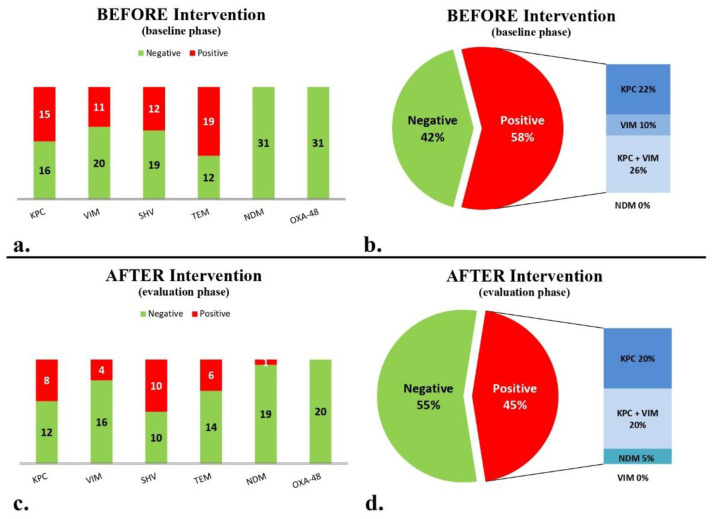
Presence/absence of carbapenemase and other β-lactamase genes of *bla*_KPC_, *bla*_VIM_, *bla*_NDM_, *bla*_OXA-48-like_, *bla*_TEM_ and *bla*_SHV_: (**a**) before intervention; (**b**) negative and positive patients with the presence of at least one carbapenemase before intervention; (**c**) after intervention; and (**d**) negative and positive patients for the presence of at least one carbapenemase gene after intervention.

During the baseline period (5 months, 895 bed-days), 37 stool samples were collected from 31 patients. Molecular analysis for the presence of the AMR genes studied (Table 1) gave the following results: 15 (48.4%) patients were colonized with bacteria bearing *bla*_KPC_, 11 patients (35.5%) with *bla*_VIM_, 12 patients (38.7%) with *bla*_SHV_, 19 patients (61.3%) with *bla*_TEM_, and no patients were colonized with either *bla*_NDM_ or *bla*_OXA-48-like_ (Figure 2a). Only six (19.4%) patients were negative for the above six AMR genes. Eighteen patients (58%) carried at least one carbapenemase gene. Specifically, seven patients carried *bla*_KPC_ only, three patients carried *bla*_VIM_ only, and eight patients both *bla*_KPC_ and *bla*_VIM_ (Figure 2b).

During the maintenance phase (5 months, 923 bed-days), the second targeted molecular screening period, including 23 stool samples from 20 eligible patients showed that 8 (40%) patients, were colonized with bacteria bearing *bla*_KPC_, 4 patients were colonized with (20%) *bla*_VIM_, 10 patients with (50%) *bla*_SHV_, 6 patients with (30%) *bla*_TEM_, 1 patient with (5%) *bla*_NDM_, and no patients with bacteria bearing *bla*_OXA-48-like_ (Figure 2c). Seven (35%) patients were negative for the presence of all six targeted genes. Patients carrying at least one carbapenemase gene totaled nine (45%; Figure 2d).

Comparing the baseline and maintenance phases, there was no significant decrease in the percentage of children harboring at least one carbapenemase gene (from 58% to 45%). The number of patients found negative for all six targeted AMR genes increased from 3 out of 31 to 6 out of 20 (*p* = 0.12). Of note, the detection of *bla*_TEM_ significantly decreased between the baseline and the maintenance phases (19/31 vs. 6/20, *p* = 0.044). There was no significant decrease between the baseline and maintenance phases for *bla*_VIM_ (11/31 vs. 4/20, *p* = 0.3) and *bla*_KPC_ (15/31 vs. 8/20, *p* = 0.58), respectively.

### 3.3. Active Surveillance for Colonization: Comparing Targeted Molecular Analysis with Bacterial Cultures for Carbapenemase Genes

The comparison of molecular analysis and bacterial cultures showed that during the baseline phase, five patient samples were found positive for at least one of the AMR genes, four had no culture taken in the context of the standard of care during their hospitalization, and two had a culture taken after 2 and 4 months correspondingly, confirming the PCR result. In 14 patients, the direct PCR detection of targeted AMR genes in stools and the culture-based phenotypic analysis were in complete agreement, but for 11 additional patients, differences between the two methods were detected. None of these 11 cases with positive PCR results also had positive cultures (Table 2, baseline phase). During the maintenance phase, the two methods were in complete agreement for 14 patients, but for the remaining 6 of the 20 patients, stool samples were found to be positive for at least one AMR gene by the PCR method, but rectal swabs, at the same time period, were found to be negative for resistance by the culture-based analysis. Four of these patients had positive cultures later, verifying the original positive PCR result; one of them had no other culture taken until discharge, and one had a single subsequent culture, which was also found negative (Table 2, maintenance phase).

## 4. Discussion

In this pilot study, we found that the direct implementation of the targeted and customized molecular detection of specific AMR genes (resistome) is an effective tool as part of various enhanced infection control measures to promptly recognize the bacterial resistance burden of a high-risk healthcare unit and successfully evaluate intervention components. The selection of these specific resistance genes was made based on the genes considered endemic in our country and in the particular hospital under study, as they represent the most common mechanisms of multidrug resistance to last-line antibiotics. Since the horizontal gene transfer of resistance genes between different organisms is proven, we are interested in identifying these genes, regardless of the organism that carries them [6,7,8]. The molecular approach we used to detect those specific AMR genes seems to be superior to the culture-based phenotypic analysis, which is commonly used in active surveillance programs in clinical settings, and in combination with the enhanced and tailored infection control measures it has a positive effect on the bacterial resistance burden in a high-risk healthcare unit.

The implementation of active surveillance using a rapid and targeted molecular tool that we developed was successfully conducted, giving the benefit to cohort patients before the development of an infection. Furthermore, the results of the rectal swab samples, taken in terms of enhanced active surveillance and processed with culture-based phenotypic methods used as a routine method by the hospital, were compared to the results of the culture-independent PCR-based metagenomics analysis used by this study. In many circumstances, cultures negative for carbapenem-resistant bacteria were accompanied by positive PCR findings for at least one carbapenemase gene. Of note, two patients in the baseline phase and four in the maintenance phase, despite initially having given a negative culture result, at a later stage they acquired a positive result for the resistance phenotype, which was detected when reculturing the same stool samples. This discordant phenomenon may be explained by the higher sensitivity and specificity of PCR methods instead of phenotypic methods, as other studies have also shown [9,10]. In addition, the metagenomic method used in this study allowed us to detect resistance genes present at the level of the microbial community of the stool sample and not only at the level of an organism that was able to grow in culture, which is an approach that has been increasingly adopted [11].

Two other important advantages of the molecular surveillance method evaluated in this study are the much shorter time needed with obvious benefits for the prompt implementation of infection control measures and the precise targeting of AMR genes included in the variety of genes searched according to the local AMR epidemiology. The result of this AMR gene targeting is huge in minimizing the costs of infection control in hospitals. Our previous experience on local epidemiology for AMR using both phenotypic and molecular tests was more than helpful to customize AMR targets in this study based on their frequency and clinical significance in critically ill children [7,8,12]. This approach may reduce any redundant cost by avoiding the inclusion of all available AMR genes that may be infrequent or without clinical significance for specific patient populations.

The applied EICM appeared to improve compliance with hand hygiene and reduce carbapenem consumption, which led to a decrease in CLABSI and, to some extent, the resistance burden in this clinical setting already endemic to MDR-B. Previous studies by our group and others have also shown that AMR incidence and infection rates diminish or are even eliminated with enhanced infection control measures enforced [5,12,13,14].

The reduction in carbapenem consumption may have contributed to the restricted spread of carbapenem resistance genes, as the correlation between the consumption of carbapenems and the resistance developed to them is highlighted by previous studies [14,15].

High levels of hand hygiene compliance, which was also confirmed by the reduced CLABSI rate, is one more contributing factor to decreased colonization. The importance of hand hygiene in the transmission of antimicrobial resistance is now well documented, and that is why global and national organizations are adopting guidelines that are constantly updated as more and more studies focus on this subject [5,16].

The direct implementation of a targeted and customized rapid molecular detection assay for searching specific resistance genes of interest according to local epidemiology in clinical samples is an effective and rapid tool to promptly recognize the burden of bacterial resistance and successfully evaluate the intervention measures applied.

## Figures and Tables

**Table 1:** Primer pair sequences, optimal annealing temperature, and the expected amplified product of indicated *bla* genes used to amplify AMR genes directly from stool samples.

Genes	Primer Pairs (5′ to 3′)	Tm Optimal	Amplicon
*bla*_KPC_	AACCTCGTCGCGGAACCATT	61 °C	785 bp
	AATCCCTCGAGCGCGAGTCTA		
*bla*_VIM_	AGCGGTGAGTATCCGACA	56.7 °C	261 bp
	ATGAAAGTGCGTGGAGAC		
*bla*_NDM_	AAGCTGAGCACCGCATTAGCC	60.2 °C	217 bp
	CGCCATCCCTGACGATCAAACC		
*bla*_OXA-48-like_	ATAAGCAGCAAGGATTTACCAA	55.5 °C	516 bp
	ACCAGCCAATCTTAGGTTCG		
*bla*_TEM_	AACTGGATCTCAACAGCGGTA	56.8 °C	510 bp
	CTGCAACTTTATCCGCCTCC		
*bla*_SHV_	CGCTTTCCCATGATGAGCACCT	61.6 °C	324 bp
	CGCCTCATTCAGTTCCGTTTCCC		

**Table 2:** Comparison of targeted molecular and bacterial culture methods among 51 patients with the detection of carbapenemase genes directly from stool samples.

	Number of Patients	
AMR Gene Detection	Baseline Phase (n = 31)	Maintenance Phase (n = 20)
Targeted Molecular Analysis	18 (58%)	9 (45%)
Initial culture-based analysis	5 (16%)	3 (15%)
Later-stage culture-based analysis	2 (6.5%)	4 (20%)

## Data Availability

We confirm that the data supporting the findings of this study are available upon reasonable request to the corresponding author due to privacy and legal reasons.
